# Use of complementary and alternative medicines for children with chronic health conditions in Lagos, Nigeria

**DOI:** 10.1186/1472-6882-8-66

**Published:** 2008-12-29

**Authors:** Kazeem A Oshikoya, Idowu O Senbanjo, Olisamedua F Njokanma, Ayo Soipe

**Affiliations:** 1 Pharmacology Department, Lagos State University College of Medicine, P.M.B 21266, Ikeja, Lagos, Nigeria; 2 Paediatrics Department, Lagos State University Teaching Hospital, Ikeja, Lagos, Nigeria; 3 Medical Student, Lagos State University College of Medicine, Ikeja, Lagos, Nigeria; 4 Academic Division of Child Health, University of Nottingham, The Medical School, Derbyshire Children's Hospital, Uttoxeter Road, Derby DE22 3DT, UK

## Abstract

**Background:**

The use of complementary and alternative medicine (CAM) is on the increase globally with a high prevalence in children and adults with chronic illnesses. Many studies have evaluated the epidemiology of medicine use for children in developing countries but none has evaluated the use of CAM for children with chronic illnesses. The aim of this study was therefore to determine the prevalence, pattern of use, parental sources of information, perceived benefits, cost, and adverse effects of CAM in children with epilepsy, sickle cell anaemia and asthma in Lagos, Nigeria.

**Methods:**

Parents of children with epilepsy (122), asthma (78) or sickle cell anaemia (118) who presented consecutively to the paediatric neurology, respiratory and haematology clinics of the Lagos State University Teaching Hospital (LASUTH), Ikeja were interviewed with a structured open- and close-ended questionnaire. The information obtained comprised the demography of both the patients and their parents; past and present treatments received by the patients; the type of CAM, if any, used by the patients; and the sources, cost, benefits and adverse effects of the CAM used.

**Results:**

A total of 303 CAMs were used by the patients, either alone or in combination witother CAM. CAM was reportedly used by 99 (31%) patients (epilepsy -38%, sickle cell anaemia – 36% and asthma – 25%). The majority (84%) of these patients were currently using CAM. The use of CAM was stopped six months prior to the study by 16 patients (16%). Biological products were the most frequently used CAMs (58%), followed by alternative medical systems (27%) and mind-body interventions (14%). Relations, friends and neighbours had a marked influence on 76% of the parents who used CAM for their children. Eighty-five (86%) parents were willing to discuss the use of CAM with their doctors but were not asked. CAM use was associated with adverse reactions in 7.1% of the patients.

**Conclusion:**

Parental use of CAMs to treat their children with epilepsy, asthma and sickle cell anaemia is common in Nigeria. Efforts should be made by doctors taking care of these patients to identify those CAM therapies that are beneficial, harmless and cheap for possible integration with conventional therapy.

## Background

The use of complementary and alternative medicine (CAM) is on the increase globally with a high prevalence in developed countries [1]. In developing countries, about 80% of the population are dependent on traditional healing methods, including herbal remedies, for health maintenance and therapeutic management of diseases [1]. In most developing countries, use of herbal remedies is relatively common and has been reported with a prevalence of 20–80% in the Caribbean [2,3], Trinidad [4,5], South Africa [6] and Nigeria [7,8]. The use of herbal remedies for young and old patients with chronic health conditions such as diabetes [9], asthma [10,11], epilepsy [7,12], sickle cell anaemia [13], hypertension [8], HIV infection and cancer [14,15], has also been widely reported.

CAM use in acutely ill children has been reported in Nigeria [16,17]; traditional/herbal medicines being the most frequently used CAMs. According to the World Health Organisation (WHO), herbal medicines are the first line of treatment for 60% of children with high fever due to malaria in Nigeria, Ghana, Mali and Zambia [18]. Cough and abdominal pains are other symptoms of children that are frequently treated with CAM in Nigeria [16,17]. Herbal CAM therapies are frequently obtained from traditional herbal medicine practitioners [19,20]. About 85% of Nigerians are known to use and consult traditional medicine for healthcare, social and psychological benefits because of poverty and disillusionment with conventional medical care [21]. Among the multitude of herbal medicines in circulation in Nigeria, only about twenty have been registered by the National Agency for Food and Drug Agency and Control (NAFDAC), and most of those are imported [22]. Only advertisements with NAFDAC endorsement are allowed in print and electronic media [22], yet aggressive strategies such as radio, television and motorcade announcements have been adopted by many unregistered CAM practitioners to market their products, which are freely available for purchase on the open market [23]. The danger of this is that misleading information is given about the herbal products and self-medication is encouraged, potentially endangering the lives of many people.

The importance of traditional medicine in Nigerian healthcare has been recognised by the national government. In December, 2006 they set up a high profile committee to develop, promote and commercialise traditional medicine products [24]. Efforts have also been made by the government to preserve indigenous Nigerian medical knowledge by boosting research into traditional medicine [24].

Extracts of plants and animals from diverse parts of Nigeria have been found to be useful for treating malaria [25,26], epilepsy [27], dementia [28], sickle cell anaemia [29-31] and bronchial asthma [32]. The potential toxicity of herbal medicines and their products due to their high levels of heavy metals [33,34], microbial contamination [35,36] and lack of safety warnings on their labels [23] are of concern regarding their use for treating children.

Complementary and alternative medicines are practices of patient treatment that are not integral parts of the conventional or orthodox medicine taught in medical schools [15]. Therefore, both physicians and patients may lack knowledge of the potential risks of adverse effects of herbal remedies [37]. Such adverse reactions are common [6,38] and may be linked to a lack of clinical trials on humans [39]. Use of CAM, specifically for chronic health conditions, has been reported to affect treatment outcome adversely [40]. Multiple therapy practices involving combined use of CAM (particularly herbal medicines) and prescribed medicines is common in some adult [41] and paediatric [10,13] populations.

Despite the abundance of studies on the use of CAM for all age groups around the world, none has explored it for children with chronic health conditions in Nigeria or other African countries. This study is therefore focused on the prevalence, pattern of use, parental sources of information, perceived benefits and cost of CAM for children with epilepsy, sickle cell anaemia and asthma. The adverse effects of CAM were also evaluated.

## Methods

This prospective study involved 318 paediatric patients with epilepsy (122), asthma (78) and sickle cell anaemia (118). These patients were recruited from the paediatric neurology, respiratory and haematology clinics of the LASUTH over a three month period (April to June 2007). Each clinic is held once in a week (respiratory clinic on Mondays, haematology and neurology clinics on Thursdays). On average, 50–60 patients are seen per visit at each of the haematology and neurology clinics, while fewer patients are seen per visit at the respiratory clinic.

The study was approved by the ethics committee of the hospital. The study instrument was a structured interview-administered questionnaire.

### The Questionnaire

The questionnaire (Additional file 1) was developed from previous studies on CAM use in paediatric and adult patients with chronic illnesses, especially those with epilepsy, sickle cell anaemia and asthma [2-8,10,11,13]. It was used to obtain the following information: demography of both the parents and their children with epilepsy, sickle cell anaemia or asthma; past and present treatments received by the patient; and the type of CAM, if any, used by the patient. Information was also obtained on the sources, cost, benefits and adverse effects of the CAM used. The questions asked were both open- and close-ended. CAM use was considered as the use of either a complementary or an alternative therapy according to the classification by the National Institute of Health [42].

The list of biological CAM (aloe vera, Forever Living^® ^products, GNLD^® ^products, Jobelyn^® ^products, Tianshi^® ^products, Yem-kem^® ^products, ginger, lemongrass, and ginseng products) used in this study was obtained from previous studies evaluating CAM use in Nigeria [7,8], and from community pharmacies and CAM practitioners in Lagos. Samples of the biological CAM products and photographs of the different alternative medical practices in Lagos, Nigeria (body scarification, charm wearing, ritual sacrifice, concoction, Chinese medicine, homeopathy, Ayurveda, and bone setting) were shown to the parents during the open-ended interview to remind them of their child's CAM use and prompt their responses. The mind-body system therapies listed in the questionnaire (spiritual healing/prayer, visualization, meditation, hypnosis and divination/incantation) were also explained to the parents during the open-ended interview.

Patients who had used CAM at least once in the last six months during present illness were regarded as CAM users; those who had not used CAM at all for the present illness were considered non-users; and those who had used CAM for the present illness but not during the previous six months were considered CAM-exposed.

The questionnaire was initially pre-tested at the oncology clinic amongst patients with childhood tumours. Modifications were made, based on the observations of the parents.

### Patient recruitment

The patients were selected randomly on consecutive presentation to the clinics so as to avoid interviewing a patient twice or more. Only their parents were interviewed; other caregivers were excluded because they may not have been able to give a proper history of the child's illness. Critically ill patients were also excluded so as not to delay care. Other inclusion criteria were proper diagnosis of the illness in the patient and documentation of the result in the case file (epilepsy diagnosed by EEG; sickle cell anaemia confirmed by genotype test; and asthma with a good history, significant findings on physical examination, and a lung function test that was in keeping with asthma), and regular clinic attendance for at least six months. The study was explained to parents who agreed to participate. They were assured that the information tendered during the interview would be treated with utmost confidentiality and that their decision to participate in the study or otherwise would not influence the treatment their child would receive.

Parents were interviewed in the waiting room where the vital signs of their children were taken. Each mother was interviewed by one of the researchers (KAO and IOS) or the research assistant, after the contents of the questionnaire had been explained to them in the native language (illiterate parents) or English (literate parents). Parents were classified according to their monthly income as low- (less than ₦ 20,000 i.e < US$170 at ₦118 = US$ 1), medium- (between ₦ 20,000 – ₦ 200,000 i.e <US$170–1,700) or high- (above ₦ 200,000 i.e > US$ 1,700) income earners.

Data were analysed using SPSS 13. Chi-square analysis was used for association between categorical variables and Student's t-test for continuous variables at a significance value of *P *< 0.05.

## Results

### Demographics of parents and their children

A total of 318 parents whose children had epilepsy (122, 38%); asthma (78, 25%) or sickle cell anaemia (118, 37%) were interviewed. The parents were predominantly female (69%), educated up to secondary school level and beyond (67%), and of low income (59%). Forty parents (13%) were not educated at all. The overall mean age of the parents was 32.1 ± 5.3 years.

The overall mean age of the patients was 4.5 ± 3.7 years. There was no significant difference in the mean age among the three groups (*P *= 0.08). One hundred and sixty nine (53%) of these children were male. The overall mean duration of illness was 3.8 ± 2.2 years (epilepsy: 4.2 ± 2.1 years, asthma: 3.8 ± 1.8 years, sickle cell anaemia: 3.3 ± 1.6 years) and the overall mean duration of clinic attendance was 23.4 ± 6.2 months.

### Current medications of the patients

Table 1 shows the list of drugs prescribed to the patients. They were all taking regular prescribed medications. Antibiotics (114, 41%), antimalarials (97, 31%) and analgesics (86, 25%) were the three medicines most frequently used by the patients in addition to their regular medicines.

**Table 1:** List of prescribed drugs taken by the patients.

**Type of medication and illness**	**Number of patient**	**Percentage of patient** **(%)**
**Epilepsy (n = 122)**		
Carbamazepine	84	69
Phenobarbitone	68	56
Valporic acid	42	34
Others		
▪ Antimalarials	32	26
▪ Antibiotics	28	23
▪ Analgesics	18	15
		
**Asthma (n = 78)**		
Salbutamol	48	62
Prednesolone	32	41
Aminophylline	25	32
Inhaler		
▪ Beclamethasone	18	23
▪ Salbutamol	14	18
▪ Salmeterol	5	6
Others		
▪ Antimalarials	17	22
▪ Antibiotics	23	30
▪ Analgesics	16	21
		
**Sickle cell anaemia (n = 118)**		
Folic acid	118	100
Proguanil	106	90
Vitamin B-complex	77	65
Ascorbic acid	65	55
Amino acid supplement	45	38
Multivitamin	41	35
Others		
▪ Antimalarials (except proguanil)	48	41
▪ Antibiotics	59	50
▪ Analgesics	52	44

### Profile of CAM utilisation

CAM was reportedly used by 99 (31%) patients (epilepsy – 38%, asthma – 25%, sickle cell anaemia -36%). Among these patients, 83 (84%) were currently using CAM (users). The use of CAM was stopped six months prior to the study by 16 (16%) patients (exposed).

The specific CAMs used are listed in Table 2. A total of 303 CAMs (either alone or in combination with other CAM) were used by the patients. Biological products were the most frequently used CAMs (58%), followed by alternative medical systems (27%). Fifty-one patients (52%) were using more than one biological product at a time. Based on the classification of CAMs, none of the patients used energy therapy. The only manipulation and touch CAM therapy was massage, which was used for asthma (2) and sickle cell anaemia (4).

**Table 2:** Types of CAM used by patients

Types Percentage of	Frequency of use of CAM			
	Epilepsy (n = 38)	Asthma (n = 25)	Sickle cell anaemia (n = 36)	Total CAM used (%)
**Biological products**				
Ginger	18	12	15	45
Herbs	13	8	15	36
Jobelyn^® ^product	3	1	16	20
Yem-Kem^® ^product	8	3	5	16
Special diet/Nutritional				
therapy	6	5	3	14
Lemongrass	6	0	8	14
Aloe vera^®^	6	4	4	14
Forever living^® ^products	10	0	2	12
GNLD^® ^product	0	2	0	2
*Tianshi*^® ^product	2	0	0	2
				
**Total**				**175 (58)**
				
**Alternative medical systems**				
Ritual sacrifice	5	2	6	13
Black soap bath	6	1	6	13
Blessed/anointed water	5	0	6	11
Blessed/anointed oil	3	1	5	9
Bone setting	0	0	9	9
Concoction soup	4	2	3	9
Scarification of body	3	0	5	8
Wearing of charms	1	0	2	3
Homeopathy	1	1	1	3
Ayurveda	0	1	1	2
Chinese medicine	0	0	1	1
**Total**				**81 (27)**
				
**Mind- body interventions**				
Spiritual healing/prayer	15	8	12	35
Meditation	3	0	0	3
Visualisation	2	0	0	2
Hypnosis	1	0	0	1
**Total**				**41 (13)**
				
**Others**				
Massage	0	2	4	6
**Total**				**6 (2)**

Forever Living^® ^and GNLD^® ^(Golden Neo Lite Diamite) products are nutritional health supplement and mega-dose multivitamins; Tianshi^® ^products are Chinese nutritional health supplements and natural products; Jobelyn^® ^product is an immune booster nutritional supplement containing Sorghum leaf extract; Yem-kem^® ^product is for curing blood and contains Cinchona 15%, Khaya ivorensis 10%, Theobroma cocoa 35% and Colocaste antoguorum 40%.

Relatives, friends and neighbours influenced 78 (79%) of the parents to use CAM for their children. Other sources of information about CAM to the parents included the media: television, radio and newspaper advertisements (13, 13%); CAM practitioners (5, 5%); churches (2%); open market advertisements (2%); and hospital staff (1%).

Most of the patients (72, 73%) used CAM daily; a few used them weekly (15, 16%) or occasionally (9%). None of the parents disclosed to doctors that they used CAM for their children. The two main reasons for this were doctors not asking the parents (84, 84.8%) and fear of the child not being treated (15, 15.2%). However, the 84 parents who were not asked about CAM use for their children were willing to discuss such use if asked by the doctors. The average cost per month amongst the majority who used CAM every day was ₦ 8, 500 (US$ 70.1).

The parents used CAM to treat or cure their children directly (80, 80%), improve their physical condition (26, 26%) or relieve symptoms of illness (17, 17%). A significantly higher number of the sickle cell anaemic patients used CAM to improve their body immunity (sickle cell anaemia -10, asthma -1; *P *< 0.001). About half the parents (46%) felt they observed some specific benefits in their children after using CAM. These included improvement in general well being (20%), relief of symptoms of the illness (18%) and complete cure of the illness (7%). Thirty-two (32%) of the parents observed some benefits of CAM in their child that were inexplicable, while 16 (16%) observed no benefit.

CAM was discontinued six months previously by 16 patients because of adverse reactions (7), dissatisfaction (7) or lack of benefits (2). Specific adverse reactions to CAMs observed were over-sedation or hyperactivity in epileptic patients (2), frequent exacerbation of difficulty in breathing in the asthmatic patients (3), and fever, diarrhoea and vomiting in the sickle cell anaemic patients (2).

## Discussion

Most studies evaluating CAM use for children have used self-administered questionnaires, which have significant limitations such as the use of terms and concepts that are confusing to parents/caregivers, and poorer response rates from incomplete filling of the questionnaire [13,43-49]. These limitations were addressed in the present study by the researchers or the research assistants conducting the interviews, providing samples of the biological CAM products and photographs of the different alternative medical practices available in Lagos, and explaining each of the terminologies used in the questionnaire to the parents. This method of interview also eliminated incomplete filling-in of the questionnaires by the parents.

Previous studies that have reported adverse reactions to CAM therapies did not look into the chronic toxicities of the biological products [44-50]; we therefore used a re-call period of six months, which we considered sufficient for any chronic toxicity to manifest itself in the patients.

A prevalence of 31% was obtained in this study. This rate is lower than the 44%–54% previously reported for chronic childhood illnesses [10,13,46] but higher than the 12%–23% reported for children with acute or stable health conditions [44-46,48,49]. One may therefore be tempted to believe that, globally, chronic health conditions promote CAM use for children more than acute illnesses. Unfortunately, this may not be true because of variations in the definition of CAM and of differences in population size between different studies.

Biological products were the CAMs most frequently used for children in this study similar to the usages for acute and chronic illnesses reported in the UK [50] and Australia [10] but contrasting with the bioenergetic therapies (prayer, spiritual and energy healing) and chiropractic manipulations used for childhood tumours and neurological disorders in Canada [43,46] and for sickle cell anaemia in the United States [13]. This variation showed that environment probably influences the type of CAM used for children.

Many studies have reported that mega-dose vitamins are the most frequently used biological CAMs for children [10,50]. Other studies, in contrast, have reported herbal medicines as the most frequently used CAMs [44,51]. In the present study, herbal medicines and their products were the most frequently used biological products. These patients were also on regular medications prescribed at the clinic; therefore they were at risk of drug-herb interactions. The risk of adverse herb-drug interactions is likely to be higher in the 52% of patients who used more than one biological product. Drug-herb interactions have been reported in patients using both herbal CAMs and prescribed medications [52,53]. This may explain the significant life-threatening adverse reactions reported by some parents in this study. The adverse reactions to CAMs reported in other studies were few and mild [43,46,50], probably because the biological products involved were mega-dose vitamins, which are seldom associated with severe adverse reactions [54].

The influence of relatives, friends and neighbours on a patient's decision to use CAM has been reported in both adults and children [7,46,47,50,51]. The high percentage (79%) of parents influenced in this study by relatives, friends and neighbours to use CAMs for their children is comparable to the 60%–86% previously reported [50,51].

None of the parents in this study disclosed the use of CAM for their children to the doctors, in contrast to the 63% and 66% who reported CAM use in other studies [51,54]. High percentages (73%–81%) of parents who use CAM for their children have been reported to be eager to discuss such usage with their doctors [45,48,55]. A similar proportion of mothers (85%) in this study was willing to discuss CAM use for their children with doctors but did not do so because they were not asked. Doctors are therefore advised to encourage parents to discuss CAM use for their children freely during hospital visits and to be willing to initiate discussions on this issue.

Only a few studies have looked at the cost of CAM [10,46,50]. Soo *et al *[46] found no significant difference in total median cost between CAM and conventional therapies (31.7 US$ vs 50.0 US$ per month). Between £ 5 and £20 per month was expended on non-medicinal CAM for children and adolescents in Wales [53], and $A 25–400 per month for children with asthma in Australia [10]. Approximately US$ 70.1 per month was expended on CAMs by each parent in the present study. This is almost twice the median amount spent on CAMs in other studies [10,46,50]. Since most of the parents in this study were low-income earners, this appears to be significant amount and may cause an economic drain on their purses. It would have been more appropriate to compare CAM costs with conventional therapy costs for these patients, but this was not done because healthcare treatment is free for children in Lagos.

CAM therapy has been reported to be beneficial for children by some parents [10,13,43,44]. Such benefits included prevention of illness, maintenance of good health, relief of musculoskeletal pain, control of asthma symptoms, treatment of mild respiratory problems, relief of pain in sickle cell anaemia and enhancement of the immune system in cancer. It is therefore not surprising to see that 80% of parents used CAM to treat or cure their children. It should be noted that the 7% of parents who perceived their children as cured were among the 16 parents who had discontinued CAM six months previously. CAM was discontinued because the symptoms of the illness recurred in their children with exacerbation after their regular medications had been discontinued. The fact that approximately half the parents (46%) of the CAM users reported some benefits of CAM to their children, albeit non-specific, calls for clinical trials of CAMs to establish parents' claim and assess the safety of the therapy for children.

This study has focused on evaluating CAM use in children with chronic illnesses. A comparative study with a control cohort of Nigerian children with no illness would have provided more information about CAM use. This is however another limitation of the study.

## Conclusion

CAM use is a common phenomenon amongst children with epilepsy, sickle cell anaemia and asthma in Lagos, Nigeria. Parents considered CAM to be beneficial for their children and were willing to discuss its use with their doctors. Paediatric doctors should initiate discussion on CAM use during clinic visits to enable parents to make informed decisions about it. Clinical trials of the biological CAM products should be done to ensure that these products are safe for children.

## Abbreviations

CAM: Complementary and Alternative Medicine; HIV: Human Immunodeficiency Virus; WHO: World Health Organisation; EEG: Electro-encephalogram; GNLD: Golden Neo Lite Diamite.

## Competing interests

The authors declare that they have no competing interests.

## Authors' contributions

KAO conceived the study, designed the study and questionnaire, participated in interviewing the parents, reviewed the statistical analysis, and drafted the manuscript. IOS participated in interviewing the parents, performed the statistical analysis, and participated in drafting the manuscript. FON participated in the design of the questionnaire and critically reviewed the manuscript. AS participated in interviewing the parents and performed the data entry.

## Pre-publication history

The pre-publication history for this paper can be accessed here:



## Supplementary Material

Additional file 1 **Questionnaire.** The questionnaire was the study instrument used to obtain the demographics of the parents and their children and information about the types of complementary and alternative medicines used for children with epilepsy, sickle cell anaemia and asthma. Click here for file
