# Supplementary material for: Use of complementary and alternative medicines for children with chronic health conditions in Lagos, Nigeria
Source: BMC Complement Altern Med. 2008 Dec 29;8:66. doi: 10.1186/1472-6882-8-66 (PMC2628640; doi:10.1186/1472-6882-8-66)
Supplement: Additional file 1 — Questionnaire. The questionnaire was the study instrument used to obtain the demographics of the parents and their children and information about the types of complementary and alternative medicines used for children with epilepsy, sickle cell anaemia and asthma. [file 1472-6882-8-66-S1.doc]

**USE OF COMPLEMENTARY AND ALTERNATIVE MEDICINES FOR CHILDREN WITH CHRONIC HEALTH CONDITIONS IN LAGOS, NIGERIA**

**SURVEY QUESTIONNAIRE**

This study is aimed to determine prevalence of CAM use for children with sickle cell anaemia, asthma and epilepsy in Lagos, Nigeria. It is purely an academic research and do not receive financial support from any company or individuals. We request you to give the most honest answers to the questions asked in the questionnaire. We assure you that all your answers would be treated with utmost confidentiality and none would influence your child’s treatment in this hospital. The findings of this research would enable doctors to understand safety of CAM use for your child. It would also enable doctors to improve the care of your child with regards to medicine use.

An interview will be conducted with you by a member of our research team who will fill your response into the questionnaire. A list and samples of biological CAM that have been used to treat sickle cell anaemia, asthma and epilepsy in Nigeria will be provided. Also, pictures of CAM practices that have been patronised in Nigeria by patients with sickle cell anaemia, asthma and epilepsy will be shown to remind you of the CAM used by your child, in case their names have been forgotten. Please feel free to seek clarifications on any question that may not be clear to you. You may choose not respond to any of the questions if you find it uncomfortable. You may also choose to opt out of the study at anytime.

Agreeing to participate in this study is taken as your consent.

Thanks for participating.

For any enquiry, please contact the following people:

**Dr Kazeem Oshikoya**

*Paediatric Department, LASUTH or Pharmacology Department, LASUCOM, Ikeja.*

**Dr Idowu Senbanjo**

*Paediatric Department, LASUTH or Paediatric Department, LASUCOM. Ikeja.*

**Prof. Olisamedua Njokanma**

*Paediatric Department, LASUTH or Paediatric Department, LASUCOM, Ikeja.*

**DEMOGRAPHICS OF PARENTS**

1. Age in yrs (a) 16- 20 (b) 21- 25 (c) 26-30 (d) 31- 35 (e) 36- 40 (f) 41-45 (g) > 45
2. Sex (a) male (b) female
3. Level of education (a) none (b) primary (c) secondary/high school (d) university/polytechnic/college of education
4. Occupation (a) unemployed (a) trading (c) artisan (d) civil servant (e) professional job (f) small scale business (g) Others (specify) ……………………………….
5. Monthly income (a) < N 20,000 (b) N 20,000 - N 200,000 (c) > N 200,000

**DEMOGRAPHICS OF PATIENT**

1. Age in years …………………………………………………
2. Sex (a) male (b) female
3. Type of chronic illness (a) sickle cell anaemia (b) asthma (c) epilepsy
4. Duration of illness………………………………. (confirm from the case file)
5. Duration of clinic attendance…………………… (confirm from the case file)

**CONVENTIONAL TREATMENTS RECEIVED FOR THE AILMENT**

1. The medications prescribed in the hospital to control your child’s ailment include

(i)……………………………………………………………….

(ii)……………………………………………………………...

(iii)……………………………………………………………..

(iv)……………………………………………………………..

(v)………………………………………………………………

1. Apart from the medications listed above, list others that your child had used for his/her ailment?

(i)……………………………………………………………….

(ii)……………………………………………………………...

(iii)……………………………………………………………..

(iv)……………………………………………………………..

(v)………………………………………………………………

1. Among the medications listed in Q2 above, which ones are your child currently using?

(i)……………………………………………………………….

(ii)……………………………………………………………...

(iii)……………………………………………………………..

(iv)……………………………………………………………..

(v)………………………………………………………………

**COMPLEMENTARY AND ALTERNATIVE MEDICINE (CAM) USE**

1. Has your child ever used CAM for his/chronic ailment (sickle cell anaemia, asthma or epilepsy)? (a) yes (b) no
2. Is your child currently using CAM? (a) yes (b) no
3. If answer to Q2 is yes, is the CAM used with your child’s regular or other medications? (a) yes (b) no
4. Has your child ever used CAM but currently not using it? (a) yes (b) no
5. If answer to Q4 above is yes, when did he/she stop using the CAM? (a) < 1 week ago (b) < 6 month ago (c) > 6 months ago
6. Among the CAM listed below and those ones shown to you, which ones are your child currently using or have used before?

**Biological products**

Aloe vera (a) yes (b) no

Forever Living® (a) yes (b) no

GNLD® (a) yes (b) no

Jobelyn® (a) yes (b) no

Tianshi® (a) yes (b) no

Yem-kem® (a) yes (b) no

Ginger (a) yes (b) no

Lemongrass (a) yes (b) no

Ginseng (a) yes (b) no

Garlic (a) yes (b) no

Ciklavit® (a) yes (b) no

High/mega dose vitamins (a) yes (b) no

Herbal medicines (a) yes (b) no

Poly herbal tea (a) yes (b) no

Special diet and supplements (a) yes (b) no

**Alternative medical practices**

Body scarification (a) yes (b) no

Wearing of charm (a) yes (b) no

Ritual and sacrifice (a) yes (b) no

Black soap bath (a) yes (b) no

Blessed/anointed water (a) yes (b) no

Blessed/anointed oil (a) yes (b) no

Concoction (eating or drinking) (a) yes (b) no

Chinese medicine (a) yes (b) no

Homeopathy (a) yes (b) no

Ayurveda/Indian medicine (a) yes (b) no

Bone setting (a) yes (b) no

**Physical and mind-body system therapy**

Spiritual healing/prayer (a) yes (b) no

Visualization (a) yes (b) no

Meditation (a) yes (b) no

Hypnosis (a) yes (b) no

Divination/incantation (a) yes (b) no

Massage (a) yes (b) no

**Energy therapy**

Bioelectromagetics (Using Tiens machine) (a) yes (b) no

Oxygen/ozone treatment (a) yes (b) no

1. Are there other types of CAM not listed above or among those in the pictures shown that your child have used? (a) yes (b) no
2. If the answer to Q7 is yes, please give their names?

(i)……………………………………………………………….

(ii)………………………………….…………………………...

(iii)……………………………………………………………..

(iv)……………………………………………………………..

(v)……………………………………………………………….

1. How often does your child use the CAM? (a) daily (b) weekly (c) monthly (d) yearly
2. What are your sources of information about the CAM your child is using? (a) relatives (b) friends (c) neighbours (d) television (e) radio (f) newspaper (g) churches (h) open market advertisement (h) medical staff (i) CAM practitioner (j) others (Please specify)……………………………………………………….
3. Have you ever disclosed using CAM for your child to his/her doctor? (a) yes (b) no
4. If answer to Q11 above is no, what are your reasons for not disclosing CAM use to your child’s doctor?

(i)……………………………………………………………….

(ii)……………………………………………………………...

(iii)……………………………………………………………..

(iv)…………………………………………………………….

(v)……………………………………………………………….

1. What is a rough estimate of the amount you spent on CAM per month? (Please specify)……………………………………………………………………
2. What are your reasons for using CAM?

(i)……………………………………………………………….

(ii)……………………………………………………………...

(iii)……………………………………………………………..

(iv)…………………………………………………………….

(v)……………………………………………………………….

1. Has your child benefited from the CAM used? (a) yes (b) no
2. If answer to Q15 is yes, what are the benefits?

(i)……………………………………………………………….

(ii)……………………………………………………………...

(iii)……………………………………………………………..

(iv)…………………………………………………………….

(v)……………………………………………………………….

(vi)……………………………………………………………….

(vii)……………………………………………………………...

(viii)……………………………………………………………..

(ix)…………………………………………………………….

(x)……………………………………………………………….

1. If your child has ever used CAM but have stopped, what are the reasons for stopping the use of CAM?

(i)……………………………………………………………….

(ii)……………………………………………………………...

(iii)……………………………………………………………..

(iv)…………………………………………………………….

(v)……………………………………………………………….

1. Have you ever abandoned conventional treatment for CAM since your child started using CAM? (a) yes (b) no
2. If answer to Q18 is yes, what are your reasons?

(i)……………………………………………………………….

(ii)……………………………………………………………...

(iii)……………………………………………………………..

(iv)…………………………………………………………….

(v)……………………………………………………………….

1. In the last 6 months of CAM therapy, has your child ever experienced any unwanted effects? (a) yes (b) no
2. If answer to Q20 is yes, what are the unwanted effects experienced by your child?

(i)……………………………………………………………….

(ii)……………………………………………………………...

(iii)……………………………………………………………..

(iv)…………………………………………………………….

(v)……………………………………………………………….

1. Would you recommend CAM to other parents? (a) yes (b) no
2. If answer to Q22 is yes, what are your reasons?

(i)……………………………………………………………….

(ii)……………………………………………………………...

(iii)……………………………………………………………..

(iv)…………………………………………………………….

(v)……………………………………………………………….
